# Common dental diseases in children and malocclusion

**DOI:** 10.1038/s41368-018-0012-3

**Published:** 2018-03-13

**Authors:** Jing Zou, Mingmei Meng, Clarice S Law, Yale Rao, Xuedong Zhou

**Affiliations:** 1 State Key Laboratory of Oral Diseases& National Clinical Research Center for Oral Diseases & Department of Pediatric Dentistry, West China School of Stomatology, Sichuan University, Chengdu 610041, China; 2 Sections of Pediatric Dentistry and Orthodontics, Division of Growth and Development, UCLA School of Dentistry, University of California, Los Angeles, CA USA; 3 Victoria General Hospital, Victoria, BC Canada

## Abstract

Malocclusion is a worldwide dental problem that influences the affected individuals to varying degrees. Many factors contribute to the anomaly in dentition, including hereditary and environmental aspects. Dental caries, pulpal and periapical lesions, dental trauma, abnormality of development, and oral habits are most common dental diseases in children that strongly relate to malocclusion. Management of oral health in the early childhood stage is carried out in clinic work of pediatric dentistry to minimize the unwanted effect of these diseases on dentition. This article highlights these diseases and their impacts on malocclusion in sequence. Prevention, treatment, and management of these conditions are also illustrated in order to achieve successful oral health for children and adolescents, even for their adult stage.

## Catching bad bite in the act

Ensuring oral health in early childhood could prevent the development of jaw misalignment later in life. Xuedong Zhou, of China’s Sichuan University, and colleagues reviewed the latest research on how common dental conditions in children influence malocclusion, where the teeth of the upper and lower jaws do not properly align, causing problems such as overbite, where the lower teeth overlap the upper teeth, or open bite, where a space remains between the upper and lower teeth when the mouth is closed. Tooth decay, prolonged thumb sucking, trauma, and the appearance of additional baby teeth are but a few of the factors that can ultimately lead to malocclusion in adulthood. Early and adequate management of these factors will establish healthy dentition and alleviate or avoid malocclusion, the researchers conclude.

## Introduction

Malocclusion, defined as a handicapping dento-facial anomaly by the World Health Organization, refers to abnormal occlusion and/or disturbed craniofacial relationships, which may affect esthetic appearance, function, facial harmony, and psychosocial well-being.^1,2^ It is one of the most common dental problems, with high prevalence ranging from 20% to 100% reported by different researchers.^3–5^ Disparity in the recorded data may ascribe to the difference in geographic position, age of included groups, registration procedures, and others. Deep overbite, midline deviation, excessive overjet, anterior crossbite, mal-alignment, space, and open bite are frequently seen types of malocclusion in clinics. The etiology of malocclusion is multifactorial and it could occur due to hereditary factors, environmental factors or the combination of these two in the affected individuals, among which dental diseases contribute a lot. Clinic work of pediatric dentistry focuses on preventing and treating various oral diseases for child and adolescent, and management of oral health from the early childhood stage in the purpose of establishing normal dentition from eruption of the first deciduous tooth to achieving final good occlusion. This paper, mainly reviewing common dental diseases in children and their influence on malocclusion, along with prevention, treatment and management of these conditions, aims to provide a broad comprehension of management of occlusal development in dental pediatric clinics. In this article, we highlighted dental caries, pulpal and periapical lesions, dental trauma, abnormality of development, and oral habits, which are common in children and link to malocclusion to some extent.

## ECC and malocclusion

As one of most prevalent chronic childhood disease,^6^ dental caries—and its complications—is the most frequent cause to seek dental care for a child.^7^ The high incidence of caries in kids is attributable to poor diet habits and oral hygiene, along with the anatomical characteristics of deciduous teeth. The caries prevalence of 5 year old children in China was 66% and the decayed, missing and filled teeth (dmft) index was 3.5 according to results of the third national oral epidemiological report.^8^ Further statistics indicate that 97% of these carious lesions did not receive proper treatment. The American Academy of Pediatric Dentistry (AAPD) defines early childhood caries (ECC) as the presence of one or more decayed (non-cavitated or cavitated), missing (as a result of caries), or filled tooth surfaces in any primary tooth in a child 71 months of age or younger. AAPD also specifies that, in children younger than 3 years of age, any sign of smooth-surface caries is indicative of severe early childhood caries (S-ECC).^9^ Under some circumstances, dental caries and its complications in the primary dentition result in much more severe and extensive consequences than in the permanent dentition.^10^

**Fig. 1:**
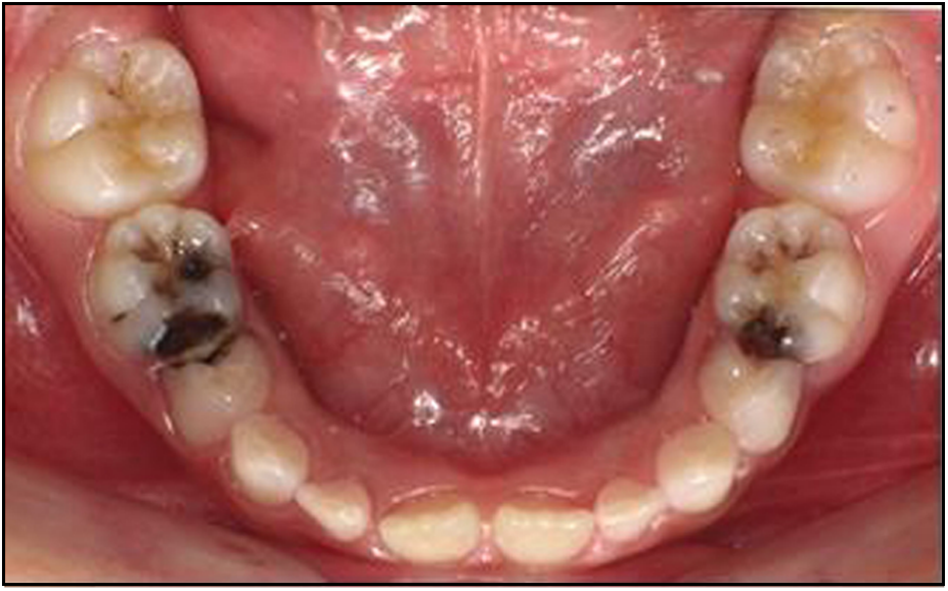
Severe decay of the first and second mandibular primary molars, intraoral arch length decreased

The extensive untreated caries and its complications, such as tooth pain, directly leads to decrease in mastication or asymmetric mastication,^11^ changing the distribution of functional occlusal contact. Long-term unilateral mastication may lead to compromised facial growth and development, resulting in malocclusion and dental-facial deformities.^12,13^ Interproximal decay of primary canines and molars may result in decreases in mesiodistal crown width. Adjacent teeth have the tendency to migrate toward the affected area, which may reduce the dental arch length (Fig. 1). Loss of arch length may lead to problems associated with tooth displacement,^14^ occlusal stability,^15^ dental crowding,^16^ chewing ability,^17^ et al.

Furthermore, caries cavitations in primary teeth may retain food debris and change the oral microenvironment. Researchers pointed out that severe caries experience during early childhood could result in a more severe caries experience during adulthood while a caries-free primary dentition has a greater likelihood to remain caries-free in the permanent dentition.^18,19^ Disintegration of the primary tooth crown from severe caries may lead to malnutrition since dental status can affect masticatory function, food preference and dietary intake, relating to developmental delays.^20^ Evidence also demonstrates a possible relationship between caries and body mass index.^21,22^ So it is of great value for pediatric dentists to prevent and treat dental caries properly and optimal restoration of the morphology and function of primary teeth is in need to maintain and improve the oral health condition of young children in clinical practice.

Multiple factors affect constant demineralization and remineralization of the tooth enamel, including bacteria (especially *streptococcus mutans*), sugar (but not sugar substitutes), saliva, and fluoride, the imbalance of which may lead to dental caries. Caries risk assessment, the determination of the likelihood of the incidence of caries, can be conducted by pediatricians to assess these key risk factors for dental caries and aid in clinical decision making regarding diagnostic, fluoride, dietary, and restorative protocols. Subjects are classified as high, moderate and low risk, and the treatment protocols are determined on that basis.^23^ Guideline on caries-risk assessment and management for infants, children, and adolescents was recommended by the AAPD Foundation, documenting biological factors, protective factors, and clinical findings in the record. Anticipatory guidance, such as dietary counseling, oral hygiene education, and application of fluoride in various forms (such as water fluoridation, fluoride toothpaste, and supplements) are adoptable to decrease the risk of dental caries and ensure the best possible health and developmental outcomes.^24^ Some novel technologies also draw our attention for the prevention and treatment of dental caries. Application of antimicrobial peptides, vaccines, probiotics, sugar substitutes and chemoprophylactic agents including classical antibiotics, plant derived compounds, anionic or cationic agents are measures taken into consideration to prevent the demineralization caused by dental biofilm. Casein phosphopeptides and fluoride are therapeutics with the ability to promote the remineralization process.^25^ For those with carious cavitations, mechanical or chemical caries removal therapy and provisional restorations are necessary in the initial control phase. More permanent restorative treatment and preventive plans are required in secondary maintenance phase.^26^ Age-appropriate strategies are advised to ensure the child’s cooperation with the examination and operative procedure. For babies and young toddlers with poor cooperation with dentists and severe dental caries in their oral cavities, treatment of ECC usually necessitates the use of general anesthesia.

## Pulpal and periapical lesions of deciduous teeth and malocclusion

**Fig. 2:**
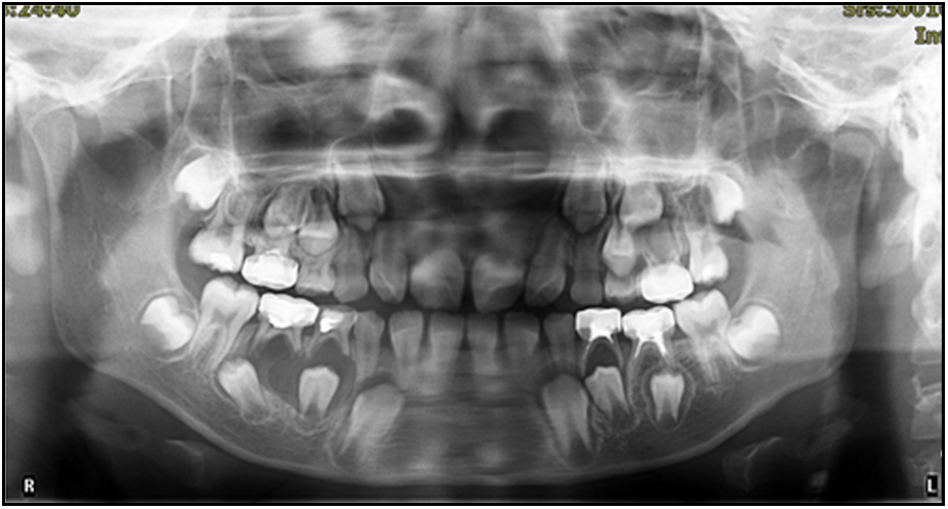
Severe periapical infection of lower right primary teeth caused displacement of lower right 2nd premolar, inducing ectopic eruption

Pulpal and periapical lesions in deciduous teeth are typically caused by oral microorganism infections of the pulp, with the most common route of entry for the microorganisms being dental caries,^27^ which may interfere normal physiological replacement. Tooth eruption and succession of primary teeth are complex process, with rather complicated mechanisms. Primary teeth interact with and depend on their successors, and vice versa. Predecessors with vital pulps are the context for normal eruption of permanent teeth and inflammation of a primary tooth pulp and penetration into the surroundings can influence the tooth germ of the permanent successor and periradicular structures if no intervention is initiated, which may arise abnormal development and eruption of the successor,^28^ such as premature, delayed or ectopic eruption of permanent tooth (Fig. 2), resulting in irregular eruption and alignment of successors and increasing the likelihood of malocclusion. Early loss of primary teeth caused by severe periapical lesions, on one hand, affects masticatory function, potentially altering maxillofacial and systemic growth and development. On the other hand, it can also lead to loss of occlusal stops and vertical dimensions, causing deep overbite and increased overjet.

In order to minimize the adverse outcomes of these endodontic diseases, dentists should make every effort to preserve the pulp vitality of the primary teeth. Guideline on pulp therapy for primary teeth was revised by AAPD.^29^ Deciduous teeth with no irreversible pulpitis can be treated with indirect pulp therapy, where no removal of the deepest carious dentin is adopted to avoid a pulp exposure. Direct pulp capping is appropriate for a primary tooth following a small mechanical or traumatic exposure other than a carious pulp exposure—with a normal pulp accompanied by a favorable response. In cases where caries removal results in pulp exposure in a primary tooth with a normal pulp or reversible pulpitis or after a traumatic pulp exposure, pulpotomy procedure can be adopted. Nonvital pulp treatment—pulpectomy is recommended for primary teeth diagnosed with irreversible pulpitis or necrotic pulp. In some subjects, where roots exhibit absorption or periapical lesions arise severe pathogenic bone destruction and harmful impact on the development or eruption of successors, there is no choice but to extract this affected primary tooth. Removable partial dentures or fixed functional space maintainers should be taken into consideration in such circumstances.

## Trauma of the primary teeth

Dental trauma is extremely common in young children, who are most vulnerable during this period owing to increased physical mobility while learning to walk and run. They are susceptible to falling and accidentally hitting hard objects. The anterior maxillary primary teeth are the most susceptible to trauma, whereas mandibular primary teeth are less prone to traumatic injury.^30^ Crown fracture, luxation, and avulsion are common types of primary tooth injury recorded.

**Fig. 3:**
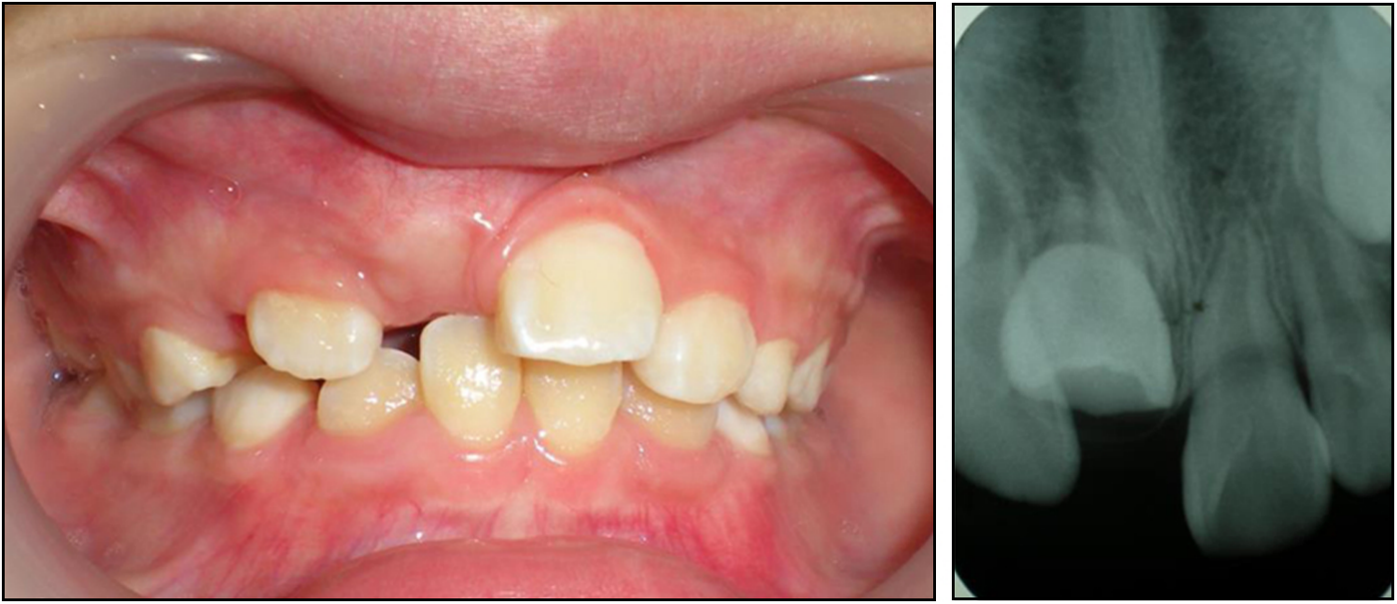
Impaction of maxillary right central incisor (left), X ray film shows the dilacerated tooth (right)

The germs of permanent teeth develop palatal to the primary teeth, in close proximity to or within millimeters of the root apices of their predecessors. Severe primary tooth trauma can cause injury or disturbance to the development of the successor permanent tooth. Partial or complete cease of root development, ectopic eruption of the permanent tooth, enamel hypoplasia, white, yellow-brown discoloration and crown-root dilacerations are potential sequelae of a traumatic incident.^31–34^ Figure 3 shows an 8-year-old boy who presented with a chief complaint of failure of eruption of his maxillary right central incisor. The patient revealed a history of trauma at the age of 3 years. He was not seen by a dentist immediately after the trauma. The primary tooth was eventually extracted when it became discolored, accompanied by gingival fistula. Radiographic result indicated that the crown and the root of permanent tooth were not on the same axis. Sometimes, Pulp necrosis and periapical lesions secondary to traumatized primary teeth are possible etiologic factors for the development of dentigerous teeth.^35^

It is of great significance to implement careful clinical and radiographic examination, make accurate and comprehensive diagnosis, carry out early intervention and adopt long-time follow-up by a multidisciplinary team in the purpose of taking precautions against possible sequelae happening to the permanent tooth caused by trauma of the deciduous teeth. The signs and symptoms of the patient should be carefully recorded to assess the response of the pulp and suffered surrounding tissues to the trauma and to predict any possible complications. Since the severity of the sequelae depends on the type and extent of the injury, the stage of development of the successor at the moment of trauma, and the child’s age at the time of the injury,^36^ comprehensive assessment is necessary to facilitate the final decision. Regular reexamination of the traumatized primary teeth should not be overlooked to avoid major damage resulting from pathological changes of the pulp or the periapical tissues.

## Abnormal tooth development and malocclusion

**Fig. 4:**
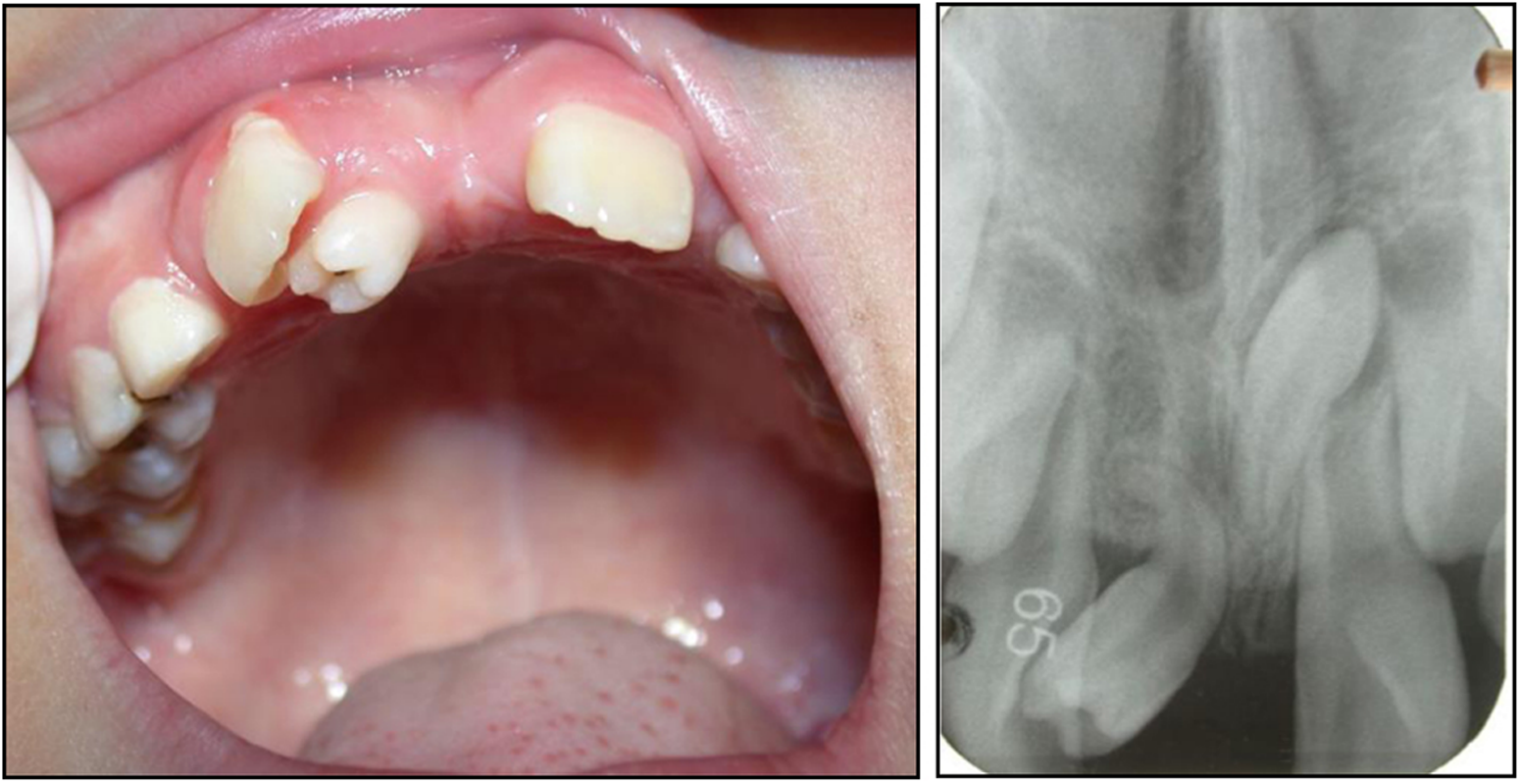
Supernumerary teeth in the area of maxillary anterior teeth resulted in a large gap and rotation of the right upper incisor; Left, intraoral photograph; Right, Radiographic illustration

Hyperdontia is an additional tooth, teeth or tooth-like structures that have either erupted or remain unerupted in addition to the regular number of teeth. Supernumerary teeth can be single or multiple, unilateral or bilateral, and evident in one or both jaws. They can develop in any region of the dental arch, but are most commonly located in the anterior maxillary region. They may erupt normally, remain impacted, appear inverted, or take an abnormal route of eruption.^37^ Supernumerary teeth frequently occur in the mixed or permanent dentition, but are rare phenomena in the primary dentition. Hyperdontia is more likely to take place in the maxilla than the mandible. Mesiodens is the most common type of hyperdontia, with the additional tooth developing between the maxillary central incisors. Some problems may be arisen from hyperdontia, including failure of eruption, crowding or abnormal diastema, displacement and/or rotation of adjacent teeth^38^ (Fig. 4), and so on.

Supernumerary teeth that have erupted into the oral cavity should be extracted in a timely manner to facilitate the eruption of the adjacent teeth and avoid displacement of the permanent teeth. For those that are impacted, treatment should be pursued in accordance with the effect on the germ of the permanent tooth. Specifically, supernumerary teeth ought to be extracted with caution if the procedure is expected to disturb the development, eruption or alignment of the permanent teeth. Otherwise, the case can be monitored carefully for extraction of the supernumerary tooth after the successor has completed root development. Cho KM^39^ published a case report involving fusion of a normal tooth and a supernumerary. Routine nonsurgical root canal treatment, bicuspidization, and appropriate restorative procedures were adopted to restore its normal occlusion.

Dental ankylosis, involving the fusion of cementum to alveolar bone, often manifests with mild to moderate progressive infraocclusion and cant of the occlusal plane. Its cause is not well defined, but may be associated with dental trauma, metabolic disturbance, genetic tendency, or local deficiency in vertical bone growth.^40^ Ankylosis of deciduous molars may influence occlusion, and impair local dental and periodontal health, such as abnormal proximal contact, food impaction, and induction of dental caries. If the ankylosed primary molar is severely infraoccluded, it may cause inclination of the adjacent teeth, supra-eruption of the opposing tooth, loss of arch-length, impaction of the succeeding permanent tooth or eruption delay, and occlusal disturbance. Restorative and prosthetic techniques are advised to restore the shape and function of infraoccluded deciduous molars. Preformed stainless steel crowns may also be indicated.^41^ When the ankylosed primary molar has no successive premolar, the decision whether to extract or maintain the ankylosed tooth requires comprehensive evaluation. Though the primary dentition is more prone to ankylosis than the permanent dentition, the influence of ankylosed permanent molars on the dentition is much more harmful than ankylosed primary molars.

**Fig. 5:**
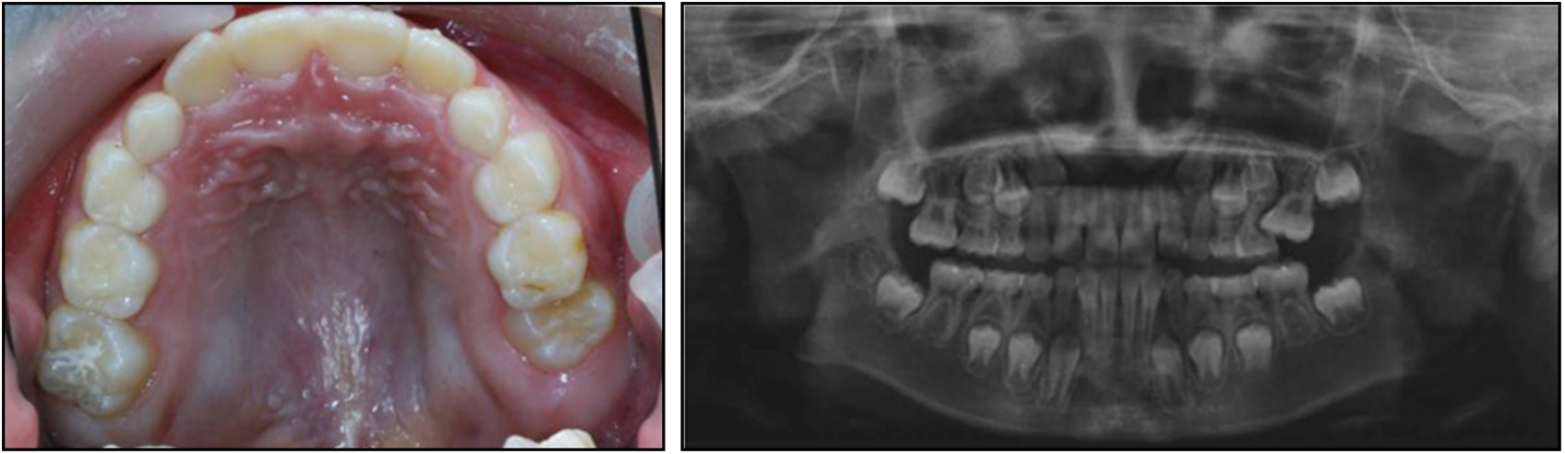
Ectopic eruption of the maxillary left first permanent molar, loss of the upper left second primary molar; Left, intraoral photograph; Right, Radiographic illustration

Ectopic eruption is defined as a tooth erupting in an abnormal position or orientation. The most commonly affected teeth in the pediatric population are the maxillary first permanent molars and maxillary canines. The occurrence of ectopic eruption of the permanent maxillary first molar (EEM) is mainly attributable to the discrepancy between the required space and the available space. Factors accounting for EEM include macrodontia in the permanent maxillary dentition, maxillary hypoplasia, and abnormal eruption angle of the maxillary permanent first molar.^42^ The prevalence of EEM varies from 0.75% to 6%.^42^ Ectopic eruption affects males more frequently than females and 66% of ectopic eruption takes place in the maxilla, unilaterally or bilaterally. EEM has a tendency to cause premature loss of maxillary deciduous second molar and decrease of the arch length (Fig. 5). It is therefore critical to correct ectopically erupting permanent molars for stable occlusion to develop. Different appliances are utilized to treat EEM, including elastomeric separator, brass wire, pre-fabricated clip separator, custom made appliances (Humphrey appliance, Halterman appliance), and the modified Nance arch appliance. Extraction of the primary molar may also be indicated.^43,44^

**Fig. 6:**
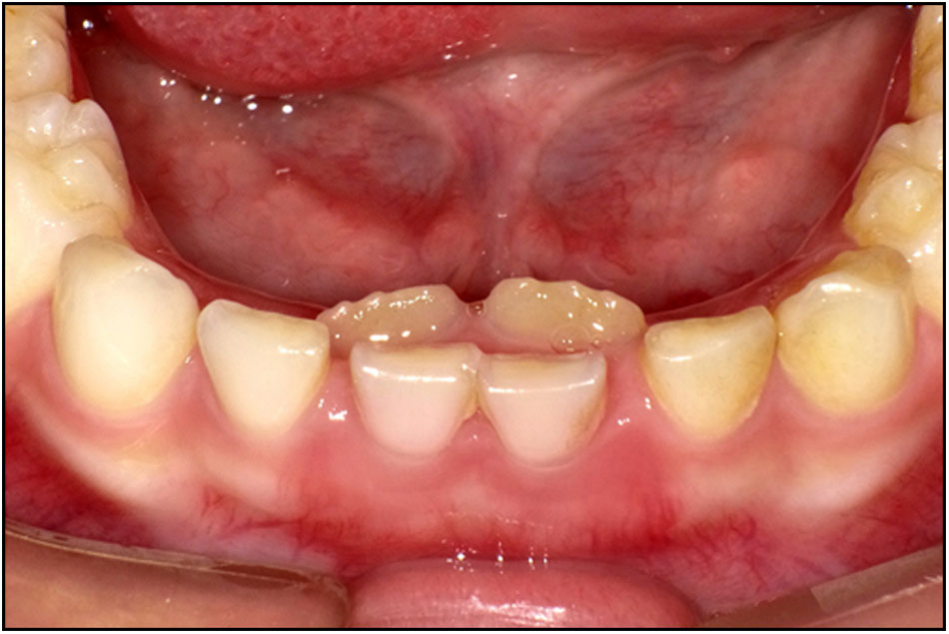
Retention of mandibular primary incisor caused ectopic lingual eruption of mandibular permanent incisor

**Fig. 7:**
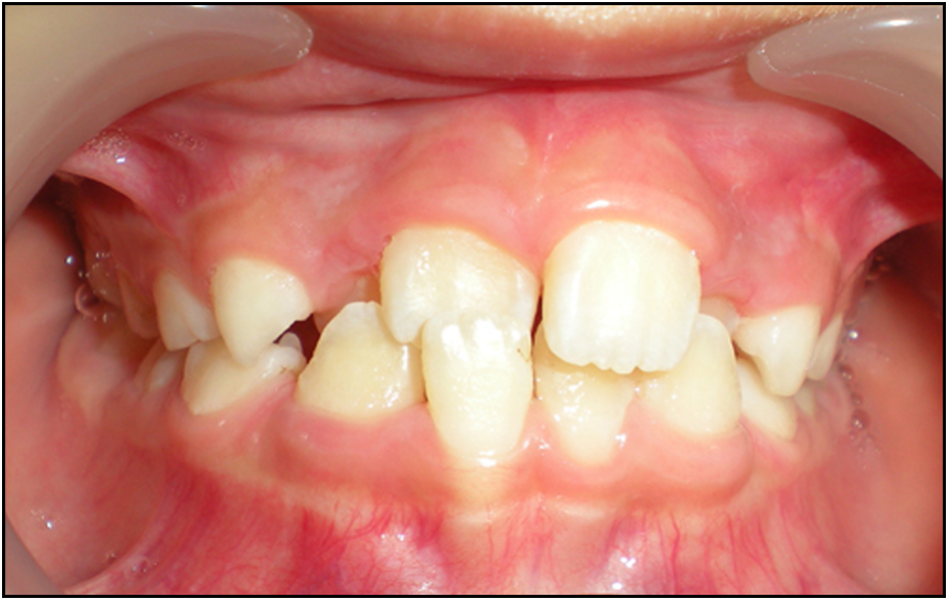
Delayed exfoliation of maxillary right primary incisor caused ectopic palatal eruption of maxillary right incisor, crossbite formed

Congenitally absent teeth can be divided into three categories based on the number of missing teeth: hypodontia, oligodontia, and anodontia. Spacing of the dental arch is the usual outcome of hypodontia. Oligodontia can result in abnormal mastication and malalignment of the dentition, and even unaesthetic appearance. Abnormal tooth morphology, such as invaginated lingual fossa, macrodontia, microdontia, and fusion may cause irregular appearance in the size and morphology of the teeth, as well as malocclusion. Over-retained primary teeth refers to the condition where the primary tooth is still retained after the successor has erupted. Prolonged retention of a primary tooth, caused by atypical root resorption or failure of root resorption can redirect the eruption path of the permanent tooth. This frequently occurs in the mandibular anterior region, where the central incisors erupt lingual to their predecessors, resulting in two lines of teeth (Fig. 6). An over-retained primary maxillary central incisor may result in ectopic palatal eruption of the permanent incisor and lead to a simple anterior cross bite (Fig. 7).

## Oral habits and malocclusion

Infants and young children frequently engage in unconscious oral habits due to some prepotential reflexes, lack of feeding, and fear or unpleasantness. A significant association of oral habits with malocclusion has been reported in various studies^45–48^ and the effect of oral habits on cranial maxillofacial growth and development is dependent on the nature, onset and duration of habits.

### Digit sucking

**Fig. 8:**
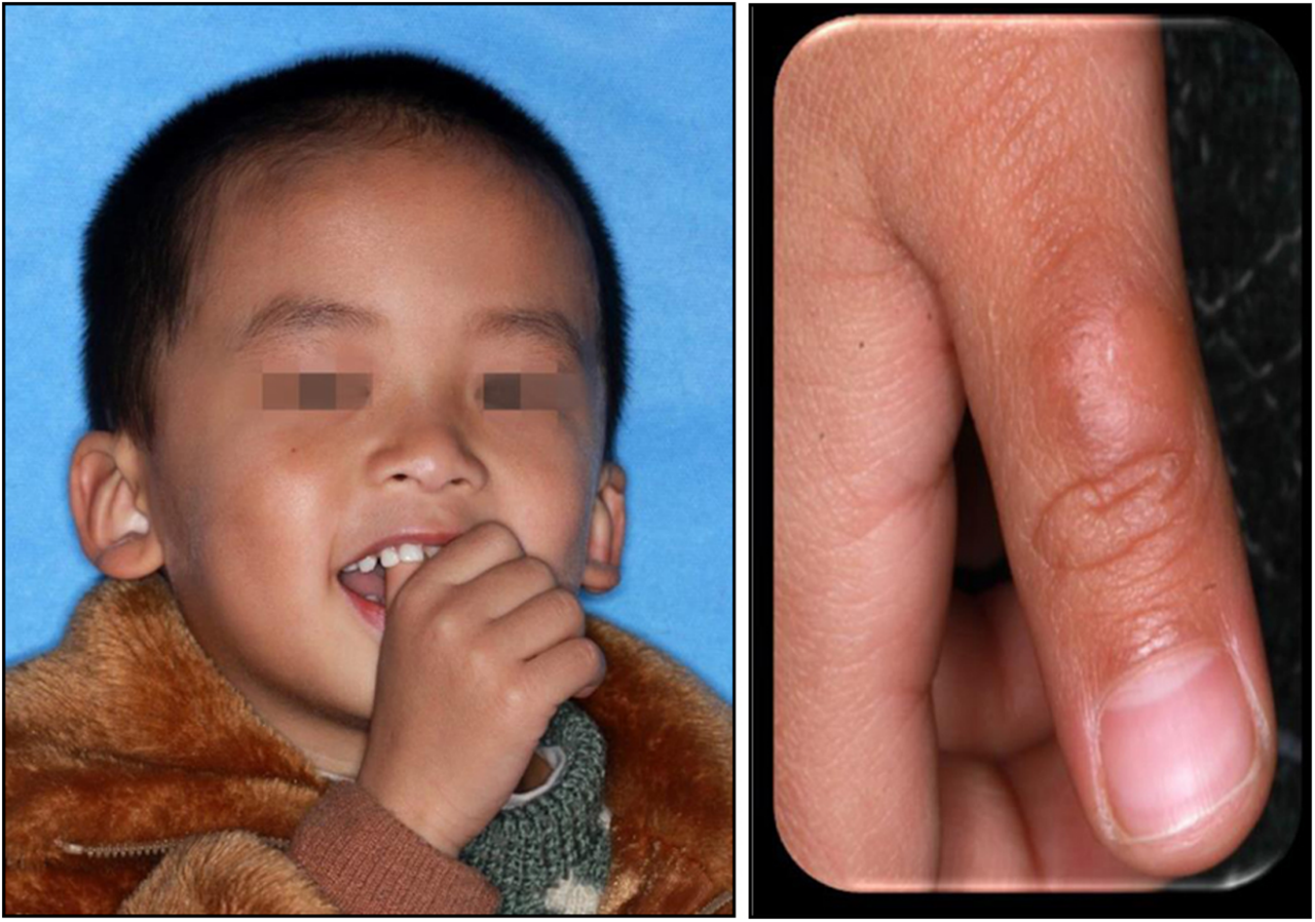
Thumb sucking and deformation of the thumb

Digit sucking, a habit occurring in childhood, can be replaced by other activities as the child matures. It includes thumb and finger sucking and may alter dento-skeletal development, leading to malocclusion if persistent over a long period of time. Individuals with this oral habit often display bite marks and deformation of the fingers or thumb (Fig. 8). Thumb sucking displaces the tongue to a low position. The change in the balance between the outward thrust of the tongue on the palate and the inward activity of the muscles of the cheeks can affect the upper arch, which frequently results in protrusion of the upper incisors and the premaxilla, atypical swallowing, anterior open bite, and posterior crossbite. The posterior teeth may extrude since the placement of the thumb between the upper and lower arch decreases occlusal contact. Downward and backward rotation of the mandible may occur. The malocclusion caused by finger sucking is different from that caused by thumb sucking. Edge to edge bite or anterior crossbite can be observed in the child with finger sucking since this behavior will guide the mandible to a forward position. Palatal cribs and arches, giving advice and incentives for changing behavior (known as psychological advice/treatment), and applying a bitter, nasty tasting substance to the children’s thumbs/fingers or combinations of these treatments can be tried to help children break this bad habit.^49^

### Tongue thrust

Tongue thrust is a condition during swallowing where the tongue gets in touch with any teeth anterior to the molars. The correlation between this habit and malocclusion is probably reciprocal, meaning that tongue habit may cause malocclusion and malocclusion might contribute to the generation of the habit.^50^ Dixit UB^51^ stated that children with tongue thrust incline to have lip incompetency, proclination of maxillary incisors, mouth-breathing habit, hyperactive mentalis muscle activity, open bite and lisping, compared with children without tongue thrust. Tongue thrust may have an influence on oral sensory perception, which can leads to a change in motor activity, exacerbating the degree of malocclusion.^52^ Surgical or orthodontic modification of the oral environment, mechanical restraints or reminders such as cribs, speech therapy, and oral myofunctional therapy can be used to motivate children to give up this habit in individualized manners.^50^

### Lip habit

Lip habit includes sucking or biting of the lips or cheeks, among which biting of the lower lip is most common. In patients presenting with lower lip sucking, strong contractions of the lower lip’s orbicularis muscle and the mentalis muscle are induced, leading to proclination of maxillary teeth and retroclination of the mandibular teeth, increased overjet, maxillary generalized spacing, mandibular incisor irregularity, and deepening of the labiomental sulcus.^53^ Upper lip sucking, on the contrary, may cause restriction of the maxillary development and anterior cross bite. It is normal to see constriction of the upper and lower arch, and posterior open bite in patients with cheek sucking and biting. A lip bumper appliance can be used to break this bad habit.^53^

### Habitual mouth breathing

Habitual mouth breathing generally occurs with obstruction of the nasal airway caused by various diseases, such as adenoid and palatine tonsillar hypertrophy, rhinitis and nasosinusitis, and hypertrophy of nasal turbinate. Alteration from nose to mouth breathing pattern affects the position of the tongue and mandible, and causes disruption in the balance of the oral and perioral muscles.^54^ Anatomic abnormality can appear in oral breathers (e.g., open bite, clockwise rotation of the mandible, increasement in the anterior lower facial height, a narrow maxilla, and a deeper palate). Children with tonsillar hypertrophy may extend their mandibles forward with the purpose of relieving dyspnea. The tongue will bring the mandible to a forward position, which can also lead to mandibular prognathism and anterior cross bite. Long-term mouth breathing can also cause gingival drying and result in accumulation of the dental plaque, with hyperplastic gingivitis as a frequent outcome. Low academic achievement and poorer phonological working memory was even reported by Kuroishi RC in children with mouth breathing, compared to participants with nasal breathing.^55^ Therefore healthcare professionals should take special note of children with mouth breathing and consider the use of vestibular shield.

### Unilateral mastication habit

Unilateral mastication habit is a phenomenon where an individual chews exclusively on one side, which can be attributable to pain caused by serious dental caries or inconvenience in chewing due to retained root tips or severely decayed crowns on the unused side. Buccal crossbite is also one reason for this oral habit. Increased lateral pterygoid muscle size has been described on the chewing side when compared the opposite side by Balcioglu HA, which may be related to the relatively high occurrence of temporomandibular disorder in children with unilateral mastication habit.^56^ Hypertrophy on the chewing side and atrophy of the non-used side can lead to facial asymmetry, unilateral cross bite and deviation of the lower midline.

New appliances have also been introduced to address malocclusion in the deciduous and mixed dentition, including the myofunctional trainer and eruption guidance appliance. Since these appliances are simple and economical, they are proposed for use in eliminating oral dysfunction, establishing muscular balance, restoring normal overjet and overbite, correcting or decreasing maxillary incisor protrusion and anterior crowding. But the cases must be carefully selected, and the operator should be well trained in their use.^57,58^

## Conclusion

Oral health management, aiming to establish a healthy dentition and alleviate or avoid malocclusion from the eruption of the first primary tooth to the accomplishment of young permanent dentition, is of great significance in the pediatric population. Dental caries, pulpal and periapical lesions, dental trauma, abnormality of development and oral habits are common diseases seen in children which hinders the establishment of normal occlusion. The issues discussed in this review will help to recognize the influence of these diseases in children on malocclusion and efforts to prevent, treat, and manage them have been discussed to pediatric dentists in face of these conditions.
